# Protein Arginine Methyltransferase 5 Inhibition Upregulates Foxp3^+^ Regulatory T Cells Frequency and Function during the Ulcerative Colitis

**DOI:** 10.3389/fimmu.2017.00596

**Published:** 2017-05-23

**Authors:** Yingxia Zheng, Liya Huang, Wensong Ge, Ming Yang, Yanhui Ma, Guohua Xie, Weiwei Wang, Bingxian Bian, Li Li, Hong Nie, Lisong Shen

**Affiliations:** ^1^Department of Laboratory Medicine, Xin Hua Hospital, Shanghai Jiao Tong University School of Medicine, Shanghai, China; ^2^Institute of Biliary Tract Diseases Research, Shanghai Jiao Tong University School of Medicine, Shanghai, China; ^3^Department of Gerontology, Xin Hua Hospital, Shanghai Jiao Tong University School of Medicine, Shanghai, China; ^4^Department of Gastroenterology, Xin Hua Hospital, Shanghai Jiao Tong University School of Medicine, Shanghai, China; ^5^Department of Anorectal Surgery, Xin Hua Hospital, Shanghai Jiao Tong University School of Medicine, Shanghai, China; ^6^Shanghai Institute of Immunology, Shanghai Jiao Tong University School of Medicine, Shanghai, China

**Keywords:** arginine *N*-methyltransferase inhibitor 1, intestinal inflammation, PRMT5, regulatory T cell, Foxp3

## Abstract

Ulcerative colitis (UC) pathogenesis is related to imbalance of immune responses, and the equilibrium between inflammatory T cells and Foxp3^+^ regulatory T cells (Tregs) plays an important role in the intestinal homeostasis. Protein arginine methyltransferases (PRMTs) regulate chromatin remodeling and gene expression. Here, we investigated whether inhibition of PRMTs affects colitis pathogenesis in mice and inflammatory bowel disease patients and further explored the underlying mechanisms. In this study, we found that protein arginine *N*-methyltransferase inhibitor 1 (AMI-1) treatments increased Tregs frequency, function, and reduced colitis incidence. Adoptive transfer of AMI-1-treated Tregs could reduce the colitis incidence. Colitis was associated with increased local PRMT5 expression, which was inhibited by AMI-1 treatment. Additionally, PRMT5 knockdown T cells produced a better response to TGFβ and promoted Tregs differentiation through decreased DNA methyltransferase 1 (DNMT1) expression. PRMT5 also enhanced H3K27me3 and DNMT1 binding to *Foxp3* promoter, which restricted Tregs differentiation. Furthermore, PRMT5 knockdown led to decreased *Foxp3* promoter methylation during Tregs induction. PRMT5 expression had a negative relationship with Tregs in UC patients, knockdown of PRMT5 expression increased Tregs frequency and decreased TNFα, IL-6, and IL-13 levels. Our study outlines a novel regulation of PRMT5 on Tregs development and function. Strategies to decrease PRMT5 expression might have therapeutic potential to control UC.

## Introduction

Protein arginine methyltransferases (PRMTs) are involved in posttranslational modification that can methylate arginine residues on histone and non-histone proteins, and this common modification has been engaged in protein trafficking, gene transcription, and tumor growth (1, 2). PRMTs can be classified as types I–IV according to their methyl arginine products and play important roles in carcinogenesis and cardiovascular and pulmonary disease (3, 4). Arginine methylation also modulates cytokine gene transcription profiles in T helper lymphocytes. Inhibition of arginine methylation impaired type 1 and type 2 helper cytokine expression levels, including IFNγ, IL-4, and IL-2 (5, 6). Recently, several small-molecule PRMT inhibitors have been synthesized, such as C21 (7). Protein arginine *N*-methyltransferase inhibitor 1 (AMI-1) specifically inhibits arginine methyltransferase activity and does not inhibit lysine methyltransferase activity (8). AMI-1 was reported that inhibits PRMT5 activity inhibits colorectal cancer growth by decreasing arginine methylation of eIF4E and FGFR3 (9). Recently, Chan-Penebre and colleagues describe the identification and characterization of a potent and selective inhibitor of PRMT5, with antiproliferative effects in both *in vitro* and *in vivo* models of mantle cell lymphoma (10). However, a full understanding of the functional role of AMI-1 in the regulation of Tregs functioning during intestinal inflammation remains unclear.

Ulcerative colitis (UC) is the primary form of chronic inflammatory bowel diseases (IBDs) and is characterized of continuous inflammation and limited solely to the mucosa and submucosa of the rectum and colon. The mechanism is related to a breakdown of immunological tolerance and that leads to pathological inflammation (11, 12). An increased number of mucosal T lymphocytes in intestinal inflammation have been clearly shown in the dextran sulfate sodium (DSS)-induced colitis model, which is more biased toward a UC case in human (13). The pro-inflammatory cytokines such as TNFα, IL-6, IL-1β are often increased in the DSS-induced colitis and UC patients. CD4^+^CD25^−^ T (Teff) can be induced into the Th1, Th2, or Th17 cells under the inflammatory condition and are important mediators of inflammation in IBD (14). Tregs are essential for the maintenance of mucosal tolerance and are capable of curing experimental colitis in animal models. The Tregs frequency has been reported to be decreased in the peripheral blood during active disease, suggesting that a numerical defect in Tregs might contribute to IBD pathogenesis (15–17). Veltkamp et al. reported that anti-TNFα therapy could decrease Tregs apoptosis in chronic IBD (18). However, the molecular mechanisms of Tregs number reduction in UC patients requires further exploration.

Foxp3 is the lineage fate-determining factor of naturally occurring Tregs (nTregs), and Tregs with the high immunosuppressive activity needs high level of Foxp3 expression and its stability (19, 20). Epigenetic regulation of the *Foxp3* locus plays important role in the stability and functionality of Tregs, and demethylation of CpG islands in the region of *Foxp3* locus-induced Foxp3 gene expression (21, 22), whereas histone 3 lysine 27 trimethylation (H3K27me3) is considered to the transcriptional suppression (23). IL-6 could repress the nTregs Foxp3 expression by increasing DNA methyltransferase 1 (DNMT1) binding and causing Foxp3 enhancer’s CpG methylation (22). In the induced regulatory T cells (iTregs), TCR signal-mediated DNMT1 stabilization and specific enrichment at the *foxp3* promoter leads to increased CpG methylation and inhibits foxp3 transcription, which could be antagonized by TGFβ signaling (24).

Here, we investigated the role of the protein AMI-1 on Foxp3 expression and its influence on Tregs function in a colitis mouse model and in patients with UC. Our data showed that AMI-1 administration increased the frequency and function of Tregs *in vitro* and *in vivo* that could provide a choice to pharmacologically therapy. Colitis was associated with increased local PRMT5 expression, and AMI-1 treatment significantly decreased the PRMT5 expression. The specific knockdown of PRMT5 not only increased the frequency of CD4^+^Foxp3^+^ T cells from the Tregs inducing system but also maintained the Foxp3 expression when the nTregs were stimulated with IL-6 *in vitro*. The underlying mechanism is that the blockage of PRMT5 decreased DNMT1 expression levels, which affected *Foxp3* gene expression. Furthermore, PRMT5 inhibition restored the defective Tregs frequency from the UC patients and enhanced protection from inflammatory colitis in the observed mice. Thus, targeting the PRMTs or specific knockdown of PRMT5 enhanced the Tregs levels and functions, making this a potential therapeutic strategy in the control of UC.

## Materials and Methods

### Mice

Male C57BL/6 and *SCID* mice were purchased from the Shanghai Laboratory Animal Center, Chinese Academy of Sciences (Shanghai, China). The animals were housed in the animal care facilities of the Shanghai Jiao Tong University School of Medicine, Xin Hua Hospital, under pathogen-free conditions. This study was carried out in accordance with the recommendations of Institutional Animal Care and Use guidelines, Xin Hua Hospital Committee. The protocol was approved by the Institutional Animal Care and Use Committee of Xin Hua Hospital.

### Patients

Patients with active UC (*n* = 25, 9 men, 16 women, mean age 46.3 ± 5.2 years, range 24–62 years) were included, and Tregs in peripheral blood were investigated. Healthy individuals (*n* = 20, 8 men, 12 women, mean age 32.7 ± 2.8 years, range 19–56 years) served as controls. Active UC was defined as a Lichtiger score >3.24. Study was carried out in accordance with the recommendations of Xin Hua Hospital of guidelines. All of the patients provided written informed consent to participate in the study in accordance with the principles of the Declaration of Helsinki. The protocol was approved by the Xin Hua Hospital of ethics Committee.

### Cytokine Production Measurements

The levels of IL-1β, IL-6, TNFα, and IL-13 produced in the culture media were quantified using ELISA kits according to the manufacturer’s instructions (R&D Systems).

### Flow Cytometric Analysis

For intracellular cytokine staining, cells were stimulated with 50 ng/ml PMA and 500 ng/ml ionomycin (Sigma-Aldrich) in the presence of GolgiPlug (BD Biosciences) for 5 h. Then, the cells were fixed and permeabilized with Cytofix/Cytoperm buffer, and intracellular cytokines were stained with antibodies against Foxp3, IFN-γ, and IL-2 (eBioscience). For PRMT5 staining, the cells were stained with an antibody or isotype control antibody (Abcam, 1/100 dilution) for 30 min at room temperature according to the manufacturer’s instructions. Flow cytometric analysis was performed with a FACS Canto II instrument (BD Bioscience) and FlowJo software (TreeStar).

### Immunoblot Analysis

Cells were directly lysed and subjected to 8% SDS-PAGE. Immunoblotting analysis was carried on by transferring proteins onto nitrocellulose membranes (Thermo Fisher Scientific) with a mini Trans-Blot apparatus (Bio-Rad). After 2 h of blocking, antibodies against PRMT1, PRMT5, DNMT1 (Abcam), and β-actin Ab (Sigma-Aldrich) were used to visualize the corresponding proteins.

### Real-time PCR

Total RNA was isolated from cell pellets using an RNeasy Mini Kit (Qiagen), and first-strand cDNA was subsequently synthesized with the Sensiscript RT Kit (Qiagen) according to the manufacturer’s instructions. Real-time PCR was performed using SYBR Green PCR Master Mix (Applied Biosystems). The data were collected and quantitatively analyzed on an ABI Prism 7900 Sequence Detection System (Applied Biosystems). The results are described as fold changes relative to mouse β-actin expression. The PCR primer pair sequences are shown in Table S1 in Supplementary Material.

### Mouse Treg Differentiation *In Vitro*

CD4^+^CD25^−^CD45RB^hi^ naïve T cells were purified with MACS columns (Miltenyi) from C57BL/6 mouse spleens. Then, they were activated under Tregs polarizing conditions (2 µg/ml plate-bound anti-CD3, 1 µg/ml soluble anti-CD28, TGFβ, 2 ng/ml, IL-2, 10 ng/ml) in the presence of T-cell-depleted splenocytes, which were utilized as the feeder cells. Finally, different AMI-1 concentrations were added to the culture system. After 3 days of culture, the cells were analyzed for Foxp3 expression.

### Isolation of Mouse Treg and Teff Cells

CD4^+^ T cells from mouse spleen were purified using the CD4^+^ T Cell Isolation Kit II (Miltenyi Biotec). Then CD4^+^CD25^−^ (Teff) and CD4^+^CD25^+^ (2% of CD25 expression) (Tregs) T cells were sorted from purified CD4^+^ T cells by FACSAria (BD Biosciences) to a purity >98%.

### Homeostatic Proliferation

Congenic CD45.2^+^ naïve T cells in a 1:1 ratio with CD45.1^+^ Tregs were purified from PBS- or AMI-1-treated donors. Then, the cells were adoptively transferred into *SCID* mice. After 7 days, recipient spleens and lymph nodes were collected and detected the CD45.2^+^ CD4^+^ T-cell total numbers with flow cytometry.

### Colitis

C57BL/6 mice were treated orally with 3% DSS (MP Corporation) and were intraperitoneally injected with 200 mg AMI-1/kg or PBS alone daily for 7 days. Then the mice weight loss, fecal blood, and stool consistency were monitored. For the T-cell transfer model of colitis, *SCID* mice were intravenously injected with 1 × 10^6^ sorted naïve T cells alone or in combination with 5 × 10^5^ Tregs specifically treated with PRMT5 shRNA or control shRNA. Then, the mice were monitored for weight loss stool consistency, gross bleeding, and sacrificed 7 weeks after the experiment was initiated, as indicated. Proximal and distal colon sections were fixed in 4% buffered formalin and stained with H&E. Gross and histological inflammation including crypt architecture, degree of inflammatory cell infiltration, muscle thickening, goblet cell depletion, and crypt abscess was scored on a scale including normal colon, mild, moderate, and severe colitis. Images were acquired with a fluorescence microscope (Leica).

### CFSE Labeling

Cell suspensions were added CFSE (Sigma-Aldrich) at a final concentration of 2 µM, 37°C for 8 min and then the cells are washed three times with PBS and suspended with complete RPMI media.

### *In Vitro* Suppression Assay

CD4^+^CD25^+^ Tregs were pretreated with the different concentration of AMI-1 under the stimulation of anti-CD3 and CD28 (2 µg/ml, respectively) for 24 h, then washed three times with PBS. Then freshly isolated CFSE-labeled CD4^+^CD25^−^ (Teff) cells from naïve C57BL/6 mice were cocultured with pretreated Treg cells at the indicated ratio, under the stimulation of splenic non-CD4^+^ cells treated with mitomycin C (Sigma-Aldrich), and anti-CD3 (2 µg/ml) for 3 days. The CFSE dilution in the Teff cells was analyzed with flow cytometry.

### shRNA Knockdown

RNA interference experiments were performed using electroporation (Amaxa) according to the manufacturer’s protocol. Briefly, we mixed 2 µg mice or human PRMT5 shRNA (Origene), respectively, or control shRNA with 1 × 10^6^ T cells in 100 µl of Amaxa mix and transfected the cells *via* electroporation. After 6 h, 50 U of recombinant human IL-2 (Roche) was added to the medium for an additional 24 h. Then, we harvested the cells and purified the GFP^+^ cells for further studies. The purity of the sorted cells was >98%.

### ChIP

ChIP assays were performed using a ChIP assay kit (Millipore) with modifications. Induced Tregs (5 × 10^6^ cells) were fixed with 1% formaldehyde, and the chromatin was sonicated and pre-cleared by incubation with Protein A/G agarose/salmon sperm DNA. Then chromatin was immunoprecipitated with H3K27me3 (Abcam) and DNMT1 (Abcam) overnight at 4°C or mouse IgG monoclonal antibody followed by incubation with Protein A/G agarose/salmon sperm DNA for 1 h. The immunoprecipitates were denatured, and the DNA was purified. The amount of immunoprecipitated DNA was quantified by real-time PCR with the ABI PRISM 7500 Sequence Detection System (Applied Biosystems) using SYBR Green. The primers used for the *Foxp3* promoter locus PCR analysis were as follows: forward: 5′ to 3′, TTC CTC CCG CTC TCT GAC TCT; reverse: 5′ to 3′, AAG CGC CAG TTG TGT ACA AAT ATC.

### DNA Methylation Analysis

Genomic DNA was purified with a GenElute TM Mammalian Genomic DNA Miniprep kit (Sigma). Methylation analysis was quantified by sequencing of the genomic DNA after bisulfite conversion using the Methyl-Detector kit (Active Motif), PCR amplification, and cloning. The primer forward was 5′-TTT TAG ATG ATT TGT AAA GGG TAA AGA A and the reverse was 5′-CAA CCT AAC TTA TAA AAA ACT ACC ACA TTA TC.

### Statistics

Student’s *t*-tests were used to analyze the differences between the groups. One-way ANOVAs were initially performed to determine whether an overall statistically significant change existed before using the two-tailed paired or unpaired Student’s *t*-tests. A value of *P* < 0.05 was considered statistically significant.

## Results

### AMI-1 Treatment Increased the Tregs Frequency and Functions and Inhibited Inflammatory Cytokines *In Vitro*

**Figure 1:**
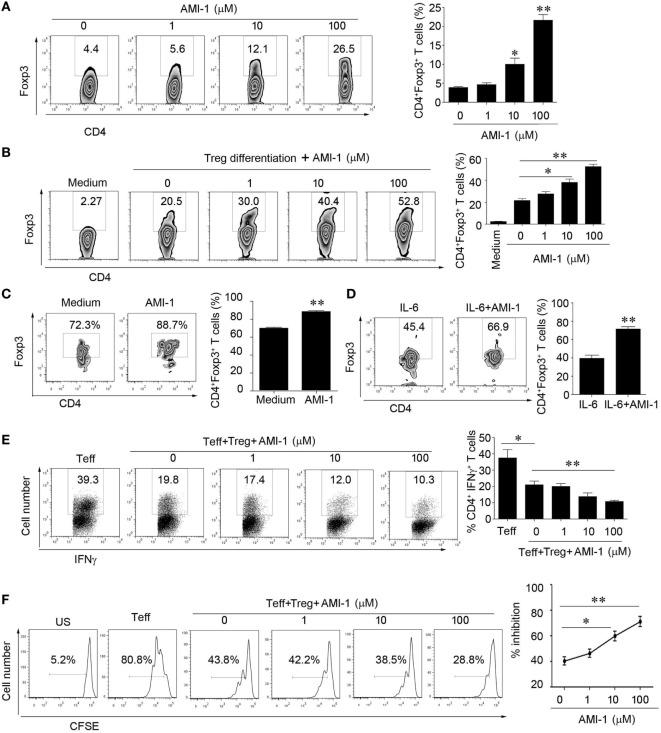
**Arginine *N*-methyltransferase inhibitor 1 (AMI-1) treatment increases the numbers and function of CD4^+^CD25^+^Foxp3^+^ Tregs *in vitro***. **(A)** Mouse splenocytes were stimulated with 1 µg/ml anti-CD3 and CD28. **(B)** Naïve CD4^+^ cells were differentiated into Tregs, and **(C,D)** FACS-sorted Tregs were stimulated with 100 µM AMI-1 or not **(C)** or 20 ng/ml IL-6 in the presence of 100 µM AMI-1 **(D)** under the stimulation with anti-CD3 and CD28 antibodies for 3 days and subjected to Foxp3 staining. The percentages of Foxp3-positive cells in the CD4^+^ T cell subset from representative subjects of three experiments were determined with flow cytometry. The data shown are means ± SEM in the right panel. **(E,F)** Tregs were treated with AMI-1 at 1–100 µM for 24 h and washed three times. The treated cells were then mixed with Teff cells at a 1:2 ratio. **(E)** The cells were analyzed for IFNγ after 48 h with FACS. **(F)** Cell proliferation was assayed with CFSE at day 4. The results are representative of three separate experiments.

The chemical form of AMI-1 utilized in this study was AMI-1 sodium salt hydrate. The primary mouse spleen cells treated with AMI-1 at concentrations up to 100 µM revealed no cytotoxicity (Figures S1A,B in Supplementary Material) *in vitro*. First, we treated the mouse splenocytes (SPs) with different AMI-1 concentrations and found that Foxp3 expressed by CD4^+^ T cells increased in a concentration-dependent manner (Figure 1A). Next, we want to demonstrate if this effect caused by the AMI-1 management was associated to Tregs differentiation, we further purified naïve CD4^+^ T cells and treated with AMI-1 or not under Tregs-polarizing conditions *in vitro*. The level of Tregs differentiation was markedly increased by the AMI-1 treatment (Figure 1B). Additionally, when FACS-sorted Tregs were cultured under the stimulation with anti-CD3 and anti-CD28, and treated with or without 100 µM AMI-1 for 3 days, then analysis of the Foxp3expression. And the results showed that treatment with the AMI-1 increased the percentage of CD4^+^Foxp3^+^ T cells than the control (Figure 1C). Furthermore, FACS-sorted Tregs were stimulated with 20 ng/ml of IL-6 and cultured with or without 100 µM AMI-1; the Foxp3 expression was elevated compared with the IL-6 stimulation alone (71.8 ± 2.6 vs. 39.6 ± 3.9%) (Figure 1D). These data demonstrated that AMI-1 treatment could increase the frequency of CD4^+^ T cells express Foxp3 from the iTreg inducing system, but also maintained the Tregs Foxp3 expression following the anti-CD3 and anti-CD28 and inflammatory cytokine IL-6 stimulation. Moreover, pretreatment of the Tregs with AMI-1 increased the Treg-mediated suppression of IFNγ secretion and Teff cell proliferation (Figures 1E,F). AMI-1 treatment in the Teff cells resulted in the expected downregulation of IFNγ secretion and cell proliferation (Figures S2A,B in Supplementary Material). We concluded that AMI-1 treatment increased the Tregs frequency and their ability to suppress cytokine secretion and the proliferation of Teff cells *in vitro*.

### AMI-1 Treatment Enhanced Tregs Suppression *In Vivo* and Ameliorated Mouse Colitis

**Figure 2:**
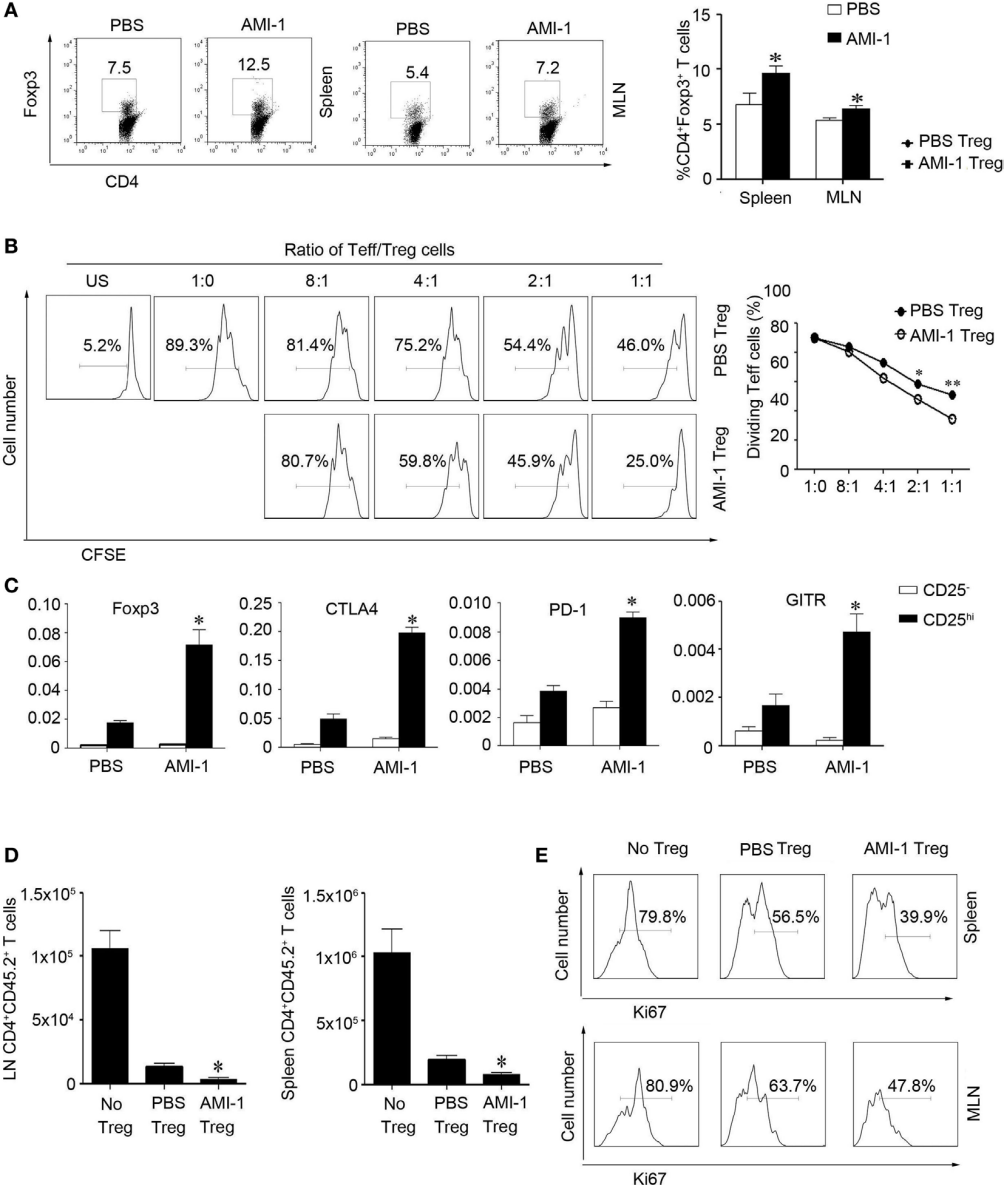
**Arginine *N*-methyltransferase inhibitor 1 (AMI-1) treatment enhanced Treg suppression *in vivo***. C57BL/6 mice received PBS or AMI-1 (200 mg/kg/day, intraperitoneal, 7 days), and then their **(A)** splenocytes (SP) and mesenteric lymph node (MLN) cells were analyzed with flow cytometry using antibodies specific for CD4 and Foxp3. The data are representative of three experiments. CD4^+^ CD25^+^ T cells (Treg) from PBS- or AMI-1 treated mice (200 mg/kg/day, intraperitoneal, 7 days) were purified, and **(B)** an *in vitro* Treg suppression assay was utilized, in which CFSE-labeled CD4^+^ CD25^−^ T cells (Teff) cells from PBS-treated mice were stimulated for 72 h with the indicated ratios of Teff to Tregs cells. The data are means ± SEM of triplicate measurements of the percentages of dividing Teff cells. The results are representative of three separate experiments. **(C)** Treg relative gene expression was analyzed with quantitative PCR. The graphs show means ± SEM. The results are representative of three separate experiments. The *P* values are for AMI-1-treated Tregs vs. PBS-treated Tregs; *P < 0.05, **P < 0.01. CD45.2^+^ naïve T cells were co-transferred with AMI-1 or PBS-treated CD45.1^+^ Tregs into *SCID* mice, **(D)** and their CD45.2^+^CD4^+^ T cells were counted after 7 days. **(E)** The SP and MLN cells were analyzed with flow cytometry using antibodies specific for Ki67. The data are representative of three experiments.

**Figure 3:**
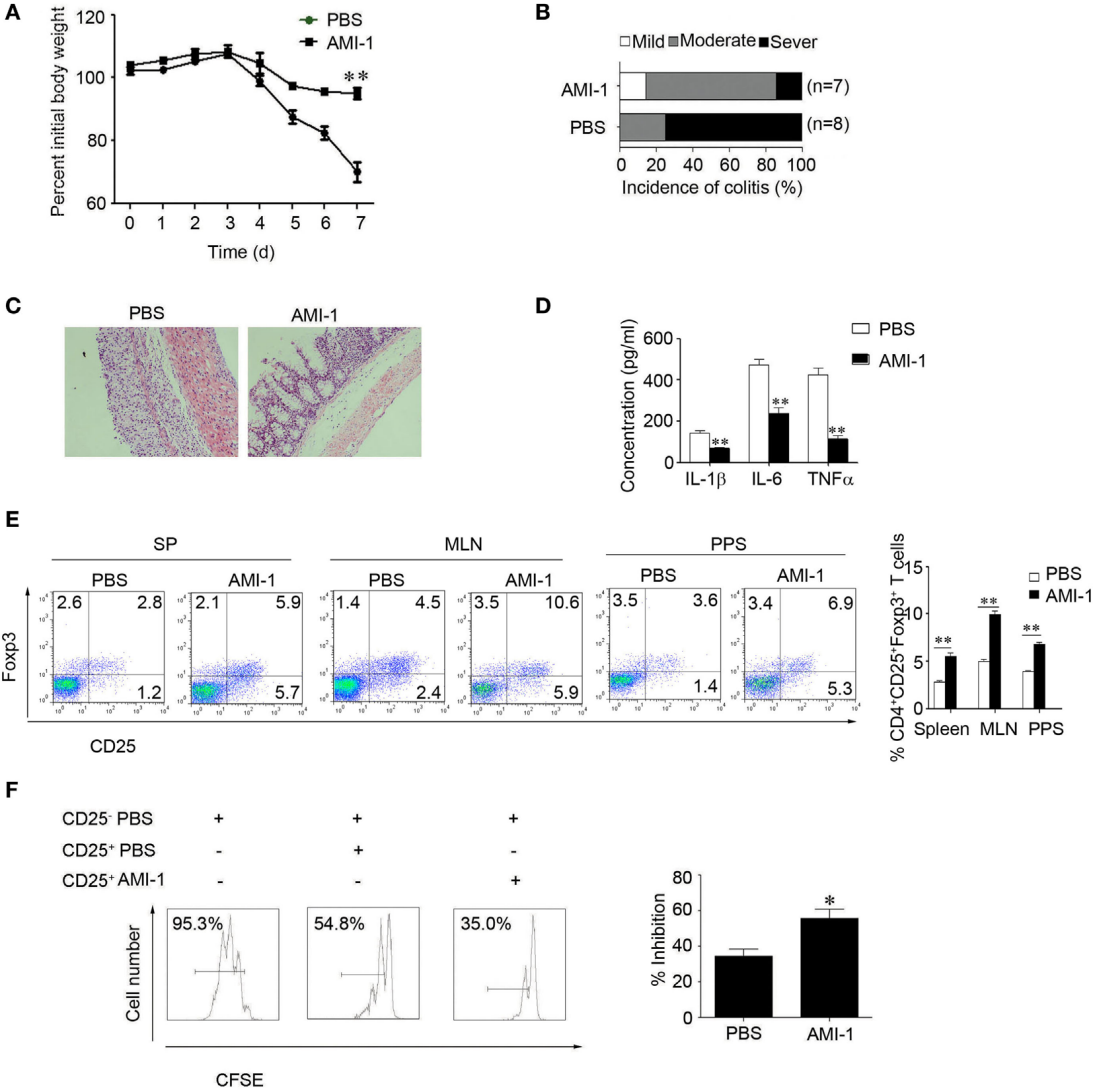
**Arginine *N*-methyltransferase inhibitor 1 (AMI-1) treatment upregulates Treg function *in vivo***. Colitis was induced in C57BL/6 mice by 3% dextran sulfate sodium. Then, the mice received PBS or 200 mg AMI-1/kg/day intraperitoneally for 7 days. Disease progression was monitored by **(A)** body weight loss, **(B)** colitis score, and **(C)** histology. The numbers in parentheses indicate the number of mice. **(D)** Colon tissue from the PBS or AMI-1 treatment was cultured for 24 h and the culture medium was collected, the levels of IL-1β,IL-6 and TNFα were measured by ELISA. **(E)** The Foxp3 expression levels in the spleen (SP) and mesenteric lymph node and Peyer’s patch cells were measured with FACS. **(F)** An *in vitro* Treg suppression assay was utilized (Treg:Teff = 1:1). The CFSE dilution in the Teff cells is shown at 72 h. Combined data from three independent experiments are presented.

We assessed the effects of AMI-1 treatment on Tregs function *in vivo*. First, we treated normal C57BL/6 mice for 7 days with 200 mg AMI-1/kg/day or PBS, and the data showed that, in the spleen and mesenteric lymph node (MLN), the frequency and absolute numbers of CD4^+^Foxp3^+^ T cells increased after the treatment of mice with AMI-1. Additionally, this treatment significantly enhanced the Tregs’ functions that can significantly inhibit Teff proliferation than control Tregs in the coculture system (Figures 2A,B). Moreover, the AMI-1-treated Tregs exhibited increased Foxp3, CTLA4, PD-1, and GITR mRNA expression levels compared with the Tregs from PBS treated (Figure 2C). Next, we transferred CD45.2^+^ naïve T cells along with AMI-1- or PBS-treated CD45.1^+^ Tregs into immunodeficient mice. After 7 days, the homeostatic T-cell numbers and proliferation level decreased under the AMI-1 coadministration in the Tregs treated with naïve T cells compared with the transfer of control Tregs with naïve T cells or naïve T cells alone (Figures 2D,E). Third, the treatment effect of AMI-1 was further investigated in the 3% DSS-induced model of colitis, in which AMI-1 (200 mg/kg/day) was administered daily for 7 days. Diminished disease severity was illustrated through gaining weight and histology performance about the colon (Figures 3A–C). To investigate the effect of AMI-1 on the production of pro-inflammatory cytokines related to DSS induce colitis, we measured the cytokines level in colon tissues. And our results indicated that AMI-1 treatment reduce the production of pro-inflammatory cytokines IL-1β, TNFα, IL-6 (Figure 3D). Additionally, the AMI-1 treatment increased the lymphoid tissue Tregs frequency and inhibition function (Figures 3E,F). Thus, AMI-1 treatment significantly increased the suppressive effect of Tregs *in vivo*.

### The Role of PRMT5 Expression in Tregs during Intestinal Inflammation

**Figure 4:**
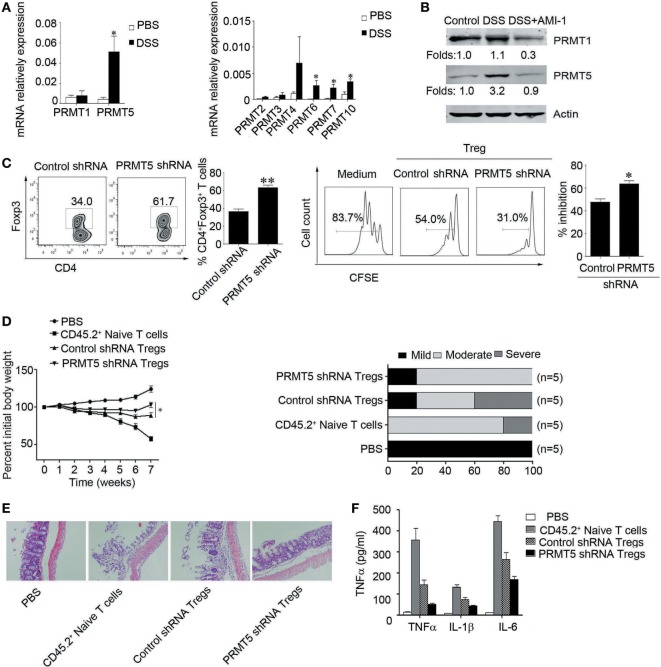
**Role of PRMT5 expression in Tregs during intestinal inflammation**. **(A)** Q-PCR analysis of PRMT expression in the colons of dextran sulfate sodium (DSS)-treated mice. The data (means ± SEM, *n* = 5/group) are expressed as the fold increase over the normal colon levels (after normalization to GAPDH). **(B)** Western blot analysis was used to detect the PRMT1 and PRMT5 expression levels in the colons of arginine *N*-methyltransferase inhibitor 1 (AMI-1)-treated DSS-induced colitis mice. **(C)** Tregs were transfected with shRNA that targeted AMI-1 or with control shRNA. The Foxp3 expression was measured with FACS (left panel), and the shRNA-transfected Tregs were mixed with CD4^+^ CD25^−^ T (Teff) cells at 1:1 ratio. The CFSE dilution of the Teff cells is shown at 72 h (right panel). *SCID* mice were injected with 1 × 10^6^ CD45.2^+^ naïve T cells, and once colitis had developed, the mice were injected with 5 × 10^5^ CD45.1^+^ Tregs transfected with PRMT5 shRNA or control shRNA. The mouse weights (mean ± SEM, *n* = 5/group) were measured at least twice weekly. *P < 0.05 to control the shRNA Tregs vs. PRMT5 shRNA Tregs. **(D–F)** The colitis scores **(D)**, representative histology **(E)**, and serum TNF-α, IL-6, IL-1β levels were analyzed at 7 weeks post-transfer of the indicated cell populations **(F)**.

As AMI-1 is a general protein arginine methyltransferase inhibitor, we detected the mRNA expression of PRMTs during the intestinal inflammation. The data show that the abundance of PRMT1 and PRMT5 expression was high in the colon tissue; however, the abundance of other PRMTs, including PRMT2, PRMT3, PRMT4, PRMT6, PRMT7, and PRMT10 expression levels, were relatively low (Figure 4A). The PRMT5 mRNA expression level was significantly increased during the intestinal inflammation (0.004 ± 0.002 vs. 0.051 ± 0.015, *P* < 0.01); however, the PRMT1 mRNA expression level was not significantly changed (0.006 ± 0.002 vs. 0.008 ± 0.005, *P* > 0.05) (Figure 4A). After the administration of AMI-1, the PRMT5 expression level was significantly reduced (Figure 4B). We also investigated the PRMT5 expression level in CD4^+^ T cells. There were no differences between the levels in Tregs and Teff cells; however, the AMI-1 treatment significantly decreased the PRMT5 expression level in both cell types (Figure S3 in Supplementary Material). When the Tregs had their PRMT5 levels specifically knocked down by shRNA (Figure S4 in Supplementary Material) and stimulated with the inflammatory cytokine IL-6, the Foxp3 expression level and the Tregs inhibition function were maintained compared with the control shRNA treatment (Figure 4C). Additionally, when the CD4^+^CD25^−^ T cells had their PRMT5 levels specifically knocked down, the IFNγ and IL-2 expression levels were significantly decreased compared with the control shRNA (Figure S5 in Supplementary Material).

To directly assess the contribution of PRMT5 to Tregs function during T cell-induced colitis, *SCID* mice were adoptively transferred with 1 × 10^6^ CD45.2^+^ naïve T cells isolated from the mesenteric lymph nodes of WT C57BL/6 mice. Once colitis developed, the mice were intravenously injected with 5 × 10^5^ PRMT5 knockdowns or control CD45.1^+^ Tregs or with the same number of CD45.2^+^ naïve T cells and followed for 3 weeks. The PRMT5 knockdown Tregs were significantly more effective than the control Tregs in promoting weight gain and reducing diarrhea (Figure 4D). The histologic analysis showed that when the colons from the mice-receiving CD45.2^+^ naïve T cells displayed dense inflammatory infiltrates, the mice-receiving CD45.2^+^ naïve T cells plus control Tregs exhibited considerably less leukocyte infiltration. The transfer of PRMT5 knockdown Tregs led to further improvement with only minor colonic infiltrates (Figure 4E). Additionally, the elevated TNFα, IL-6, IL-1β levels were significantly attenuated in the mice that received PRMT5-specific knockdown Tregs compared with the control Tregs (Figure 4F). These data from the colitis model indicated that Foxp3^+^ Tregs lacking PRMT5 exhibited increasing suppressive functions *in vivo*.

### PRMT5 Knockdown T Cells Exhibit Increased Sensitivity to TGF-β

**Figure 5:**
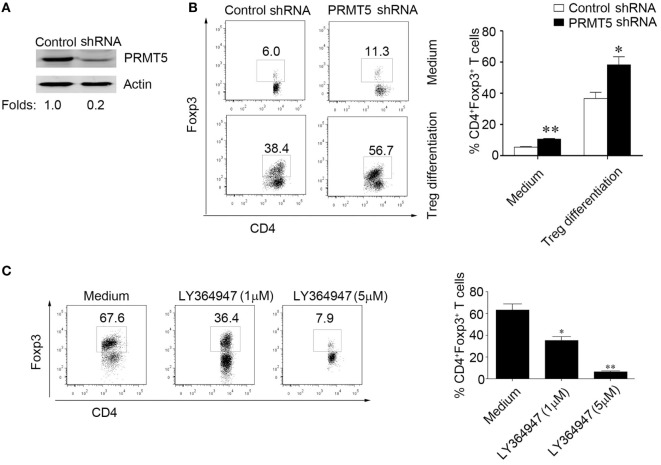
**PRMT5 knockdown in T cells reveals increased sensitivity to TGF-β**. CD4^+^CD25^−^ T cells were transfected with shRNA that targeted PRMT5 or with control shRNA. **(A)** After 48 h, the PRMT5 expression was measured with Western blot analysis. **(B)** The transfected cells were or were not differentiated under Treg-promoting conditions and were analyzed at day 3 for Foxp3 expression with flow cytometry. The numbers represent the CD4^+^Foxp3^+^ cell frequency. **(C)** CD4^+^CD25^−^ T cells were transfected with PRMT5 shRNA, and they were differentiated under Treg-promoting conditions in the presence of a TGF-β signaling inhibitor (LY364947, 1 and 5 µM). The data shown are representative of at least three independent experiments. The *P* values represent significant differences between the LY364947 and medium control-treated cells. *P < 0.05, **P < 0.01.

Data show significantly augment proportions of Foxp3^+^ cells when naïve T cells were PRMT5 knocked down and treated with TGF-β compared with control shRNA T cells (Figures 5A,B). And treatment with different concentrations of a chemical inhibitor LY364947, which inhibited TGF-β signaling, significantly reduced the Tregs frequency in the Tregs differentiation condition cell culture from the PRMT5 knockdown naïve T cells (Figure 5C). These data show that PRMT5-specific knockdown T cells strength sensitivity to TGF-β and result in increasing Tregs frequency.

### The Role of PRMT5 in the Development of Foxp3 Gene Silencing

**Figure 6:**
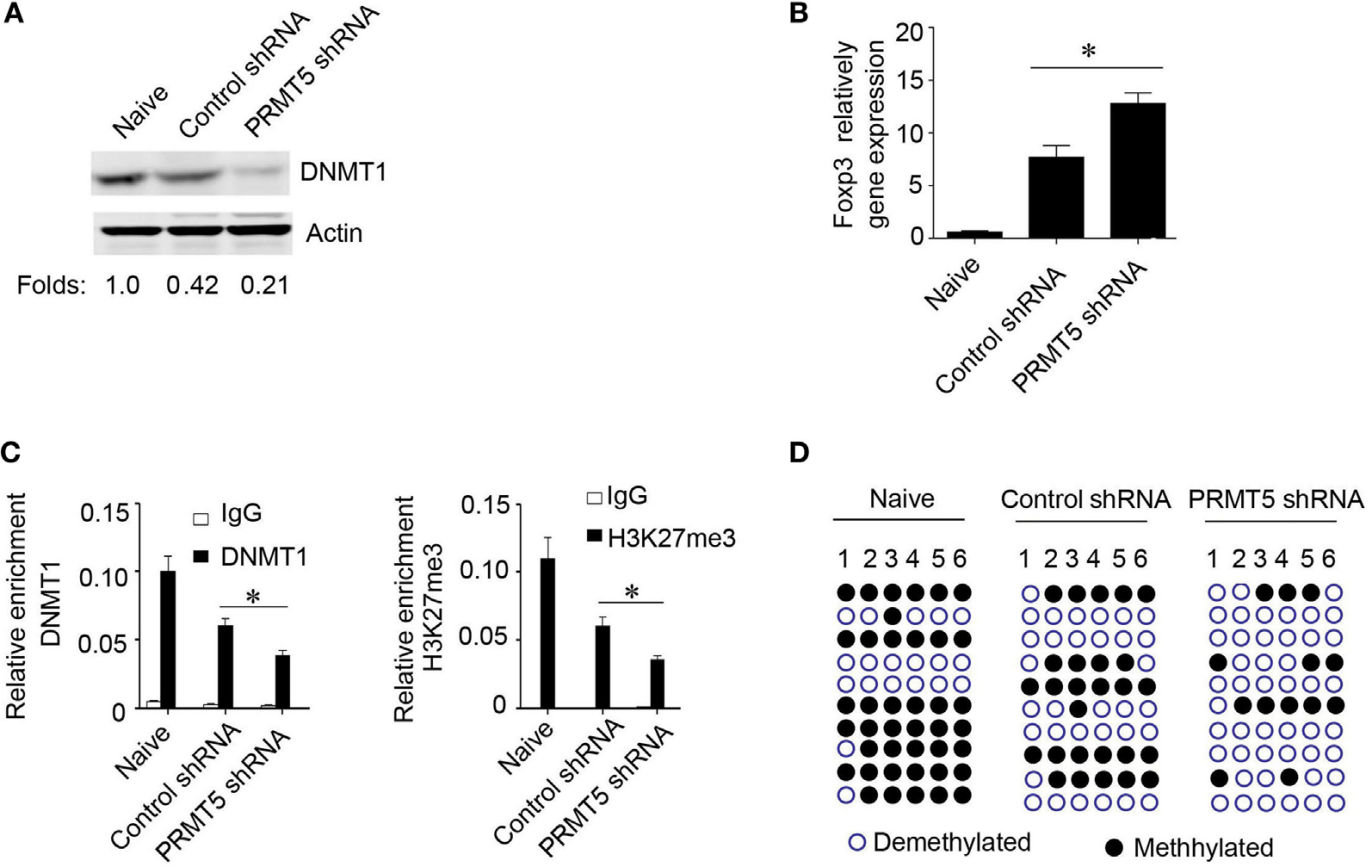
**Role of PRMT5 in developmental *foxp3* gene silencing**. **(A–D)** CD4^+^CD25^−^ T cells were transfected with PRMT5 shRNA and differentiated under Treg-promoting conditions. **(A)** After 48 h, the DNA methyltransferase 1 (DNMT1) expression levels were measured by western blotting. **(B)** Foxp3 gene expression levels were analyzed with quantitative PCR. **(C)** ChIP analysis was utilized with antibodies specific for DNMT1 (left panel) or H3K27me3 (right panel). Quantitative PCR was performed using primers for the promoters of the specific genes. The data shown are from three independent experiments. Statistics were utilized to compare differentiated cells with naïve T cells. *P < 0.05. **(D)** Analysis of individual CpG sites within the *Foxp3* promoter by bisulfite conversion, cloning, and sequencing. Open circles: unmethylated CpGs; filled circles: methylated CpGs. The data represent three independent experiments.

We further explored how PRMT5 mediated the observed *foxp3* gene expression. PRMT5 has been reported as a type II arginine methyltransferase and could silence gene expression by symmetrical di-methylation of H4R3, H2R3, and H3R8 (25–27). Additionally, PRMT5 could modulate the methylation of H4S1ph, H4K20me3, H3K9me3, and H3K27me3 (28). DNMT1 serve as the key modulators that control the transcriptional accessibility to the *Foxp3* regulatory regions (24, 29). We hypothesized that PRMT5 could influence DNMT1 expression and regulate *foxp3* gene expression in naïve T cells. Data show that, in the *in vitro* system, for Tregs differentiation, when the naïve T cells were specifically knocked down of PRMT5, the DNMT1 expression level was also decreased; however, the *Foxp3* gene expression level was increased (Figures 6A,B). We next tested whether PRMT5 could affect DNMT1 binding to the *Foxp3* promoter site or the inhibitory epigenetic modification of H3K27me3 by performing a ChIP analysis using anti-DNMT1 and H3K27me3 antibodies. Both DNMT1 and H3K27me3 were bound to the *Foxp3* promoter region in naïve T cells and to a lesser extent when the T cells were in a Tregs differentiation system; however, when PRMT5 was specifically knocked down, the binding was significantly reduced (Figure 6C). As PRMT5 can modulate H3K27me3, which can bind to the *Foxp3* promoter region, we assessed the effects of PRMT5 deletion on *Foxp3* promoter methylation through bisulfite conversion, cloning, and sequencing studies. The PRMT5 knockdown cells exhibited reduced CpG methylation within the *foxp3* promoter of the T cells following Tregs induction (Figure 6D).

### Inhibition of Human PRMT5 Promotes Tregs Frequency and Function in Patients with UC

**Figure 7:**
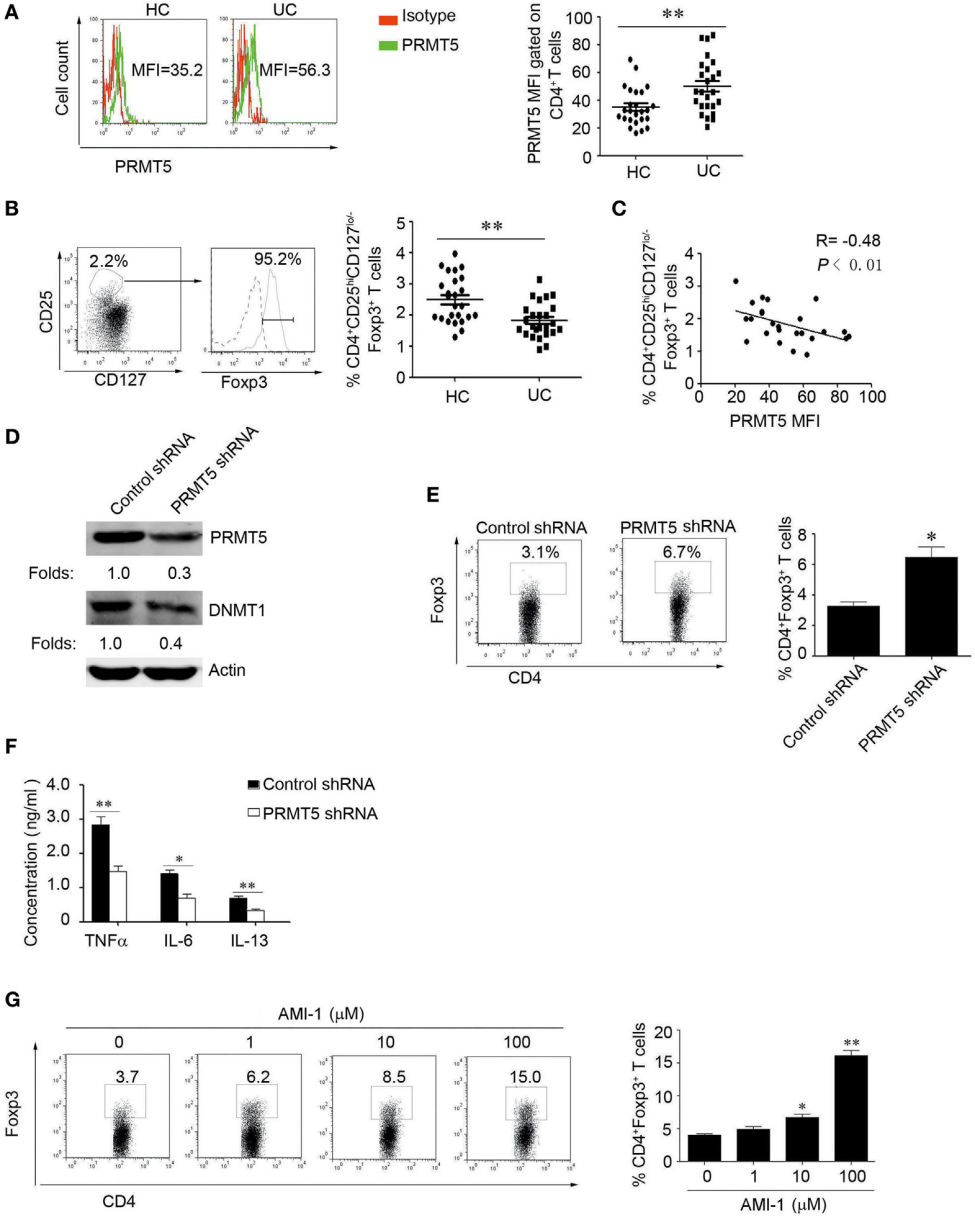
**The inhibition of human PRMT5 promotes Treg differentiation and function in ulcerative colitis (UC) patients**. **(A)** CD4^+^ T-cell subsets derived from UC patients and healthy controls (HCs) (*n* = 25 in both cases) were analyzed for PRMT5 expression with FACS. The bars represent the mean and SEM (MFI, mean fluorescent intensity). **(B)** The representative FACS data from CD4^+^CD25^hi^CD127^lo/−^ T-cells and the Foxp3^+^ expression levels are shown. The dotted line and solid line represents isotype control and Foxp3 antibody, respectively. The CD4^+^CD25^hi^Foxp3^+^CD127^lo/−^ Treg frequency from the HC (*n* = 25) and UC patients (*n* = 25) (right panel). *P < 0.05 vs. PBMCs from HCs. **(C)** Correlation of PRMT5 levels with Tregs in the UC patients (*n* = 25). The *R* value represents the calculated regression coefficient. The *P* value indicates the correlation index between the groups. **(D,E)** CD4^+^ T cells isolated from UC patients (*n* = 5) were electro-transfected with PRMT5 shRNA or control shRNA and cultured for 72 h. Western blot detect the PRMT5 and DNA methyltransferase 1 expression, actin as the loading control **(D)**. Foxp3 expression was measured with flow cytometry **(E)**. **(F)** The culture medium was collected, and the TNFα, IL-6, and IL-13 levels were evaluated with ELISAs. **(G)** CD4^+^T cells isolated from UC patients (*n* = 5) were stimulated with different arginine *N*-methyltransferase inhibitor 1 concentrations for 3 days. The percentages of Foxp3-positive cells in the CD4^+^ T-cell subset from the representative subjects were determined with flow cytometry (left panel). The data shown are means ± SEM in the right panel. The asterisks represent statistical significance between the groups, *P < 0.05; **P < 0.01.

To further explore the role of PRMT5 in Tregs development in UC patients, we first detected their PRMT5 expression levels, and they were significantly increased in the patients’ CD4^+^ T cells (Figure 7A). Consistent with previous reports, we found that the Tregs number were deficient in the UC patients (Figure 7B), and the PRMT5 expression level was negatively correlated with the Foxp3 expression level in the patients (Figure 7C). However, when the CD4^+^ T cells from the UC patients were specifically knocked down of PRMT5, the DNMT1 and Foxp3 expression level was increased compared with the control shRNA treatment (Figures 7D,E). We further detected the UC-related inflammatory cytokines levels (30) and found that inflammatory cytokines TNFα, IL-6, IL-13 were decreased in the PRMT5 shRNA group (Figure 7F). Additionally, CD4^+^ T cells isolated from UC patients were treated with AMI-1 and their Foxp3 expression levels were measured. The AMI-1-treated Tregs exhibited promoted Foxp3 expression (Figure 7G). Our data demonstrate that the PRMT5 could have a potential action on the promotion of Tregs differentiation and the inhibition of inflammation cytokines in UC patients.

## Discussion

Protein arginine methyltransferases regulate diverse cellular programs, like tumor development, RNA transportation, chromatin reconstruction, and cell cycle control. However, how the PRMTs regulate the immune system, especially in the regulation of Tregs development and function in an inflammatory disease setting has been elusive. In this study, we showed that AMI-1 administration increased the frequency of peripheral Foxp3^+^ Tregs and increased their suppressive capability. Consistent with the AMI-1-treatment increased Tregs suppressive function *in vitro*, AMI-1 administration also promoted Tregs function by inhibiting homeostatic T-cell proliferation in *SCID* mice. PRMT5 expression increased during experimental inflammation colitis and significantly decreased after the AMI-1 treatment. Thus, our study reveals significant new roles that PRMT inhibitors could regulate immune responses under inflammatory settings by PRMT5, which can modulate Tregs differentiation and function. Additionally, inhibition of PRMT5 decreased DNMT1 expression, which can induce CpG methylation, also effected H3K27me3 modification, and both of which had critical roles in the regulation of *Foxp3* gene expression. We do not propose that Foxp3 is the only target of the PRMT inhibitor in T cells, but rather that the effects of the PRMT inhibitor on Foxp3 in Tregs constitutes an important, and previously uncharacterized, therapeutically relevant mechanism of action. Recently, Liu et al. has reported that deleting PRMT5 in bone marrow cells reduced pro- and pre-B and impaired T cell development, suggesting that it is required for lymphocyte development (31). So PRMT inhibitor may involve other immune cell types, which is very interesting and required in the further study.

The pathogenesis of UC is complex and involves defects in mucosal barrier function and the immune system. Despite many important findings in recent years, it is still largely unknown why the mucosal immune response is over-reactive in UC. Reduced peripheral Tregs numbers have been associated with both active CD and UC, and they were shown to be inversely correlated with disease activity (32, 33). Collectively, our study demonstrates a new mechanism for enhanced Tregs number and functioning as a result of reducing PRMT5 expression. Studies demonstrate that PRMT5 could modulate gene expression by regulating some activities of transcription factors or histones modifications (34, 35). PRMT5 also participated in many incidents, like transcriptional inhibition of p53, which is most important tumor suppressor gene, or modulated NFκB activity and engaged in cellular transformation (36–38). PRMT5 also repressed inflammatory cytokines like IL-8 and IL-1β expression, but promoted IL-2 production and myogen expression (6, 25). However, how PRMT5 regulates Tregs fuction remains elusive. Our study revealed beneficial effects of AMI-1 therapy in inhibiting PRMT5 and decreasing DNMT1 levels. Also, the protective role of the PRMT inhibitor was analogy in PRMT5 knockdown Tregs by relieving colitis. Hence, the use of AMI-1 or PRMT5 deletion alone could ameliorate injury from inflammatory colitis.

Our data show that PRMT5 loss led to enhanced Foxp3 expression and increased Tregs function and that its reduction promoted Foxp3 demethylation and restored Tregs function to normal. Additional studies are required to further elucidate the importance and precise interactions of PRMT5 with Foxp3 using PRMT5 conditional knockout mice. A good knowledge of the interactions between PRMT5 and Foxp3 could offer new choices for developing specific small-molecule and specially targets toward Tregs, rather than using a widely used inhibitor such as AMI-1. Collectively, our study demonstrates a new mechanism that enhances Tregs number and functioning as a result of PRMT5 knockdown. We conclude that developing a small-molecule inhibitor or other strategies to inhibit the function of PRMT5 might be of therapeutic importance in the management of UC and potentially other autoimmune diseases.

## Ethics Statement

All of the patients provided written informed consent to participate in the study, which was carried out in accordance with the principles of the Declaration of Helsinki and was approved by the ethics committee of Xin Hua Hospital.

## Author Contributions

YZ, LH, and LS designed and discussed the study. YZ and LH carried on most of the experiments, collected and analyzed data. WG and MY recruited study participants and provided clinical samples. WW helped with cell sorting. YZ, LH, YM, GX, BB, LL, HN, and LS contributed to the writing of the paper.

## Conflict of Interest Statement

The authors declare that the research was conducted in the absence of any commercial or financial relationships that could be construed as a potential conflict of interest.
