# Supplementary material for: Protein Arginine Methyltransferase 5 Inhibition Upregulates Foxp3+ Regulatory T Cells Frequency and Function during the Ulcerative Colitis
Source: Front Immunol. 2017 May 23;8:596. doi: 10.3389/fimmu.2017.00596 (PMC5440547; doi:10.3389/fimmu.2017.00596)
Supplement: Supplementary file 1 [file data_sheet_1.pdf]

Protein arginine methyltransferase inhibition regulates Foxp3+ regulatory T cell function during intestinal inflammation

Yingxia Zheng, [1, 2, #] Liya Huang,[3, #] Wensong Ge, [4] Ming Yang, [5] Yanhui Ma, [1] Guohua Xie,[1] Weiwei Wang, [1] Bingxian Bian,[1] Li Li, [1] Hong Nie, [6] Lisong Shen[1,*]

Supplementary information

Cell viability

The viability of spleen cells was analyzed by CellTiter-Glo luminescent assay (Promega, Madison, WI) after treated spleen cells with different concentrations of AMI-1 according to the manufacture's introduction. In brief, splenocytes were cultured in a 96-well microplate at 1 x 10^5 cells per well and stimulated with anti-CD3 and anti-CD28 (BD Bioscience) at 1 μg/ml. Medium with AMI-1 (Sigma-Aldrich) at final concentrations of 0.1, 1, 10, 100, and 500 μM was added to all but the control wells and incubated for 72 h. After culturing, the luminesence values were measured with microplate computer software (Bio-Rad Laboratories).

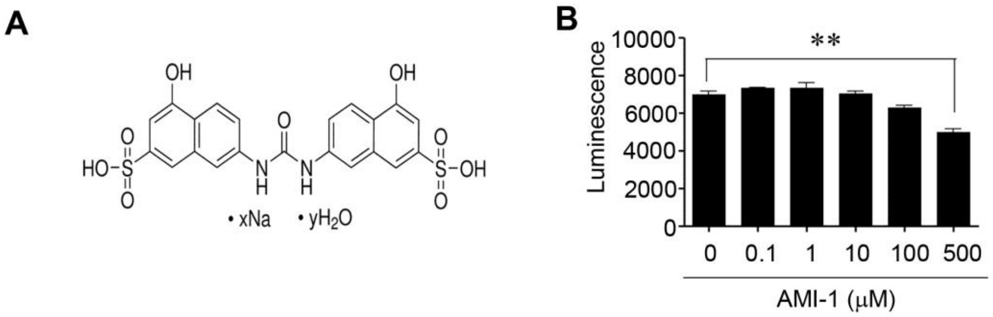

Supplementary Fig. 1: AMI-1. (A) The AMI-1 chemical structure. (B) Cell viability was evaluated, as measured by luminescence in C57/BL6 mouse splenocytes stimulated with 1 μg/ml anti-CD3 and anti-CD28 and cultured in the presence of increasing AMI-1 concentrations. **P<0.01.

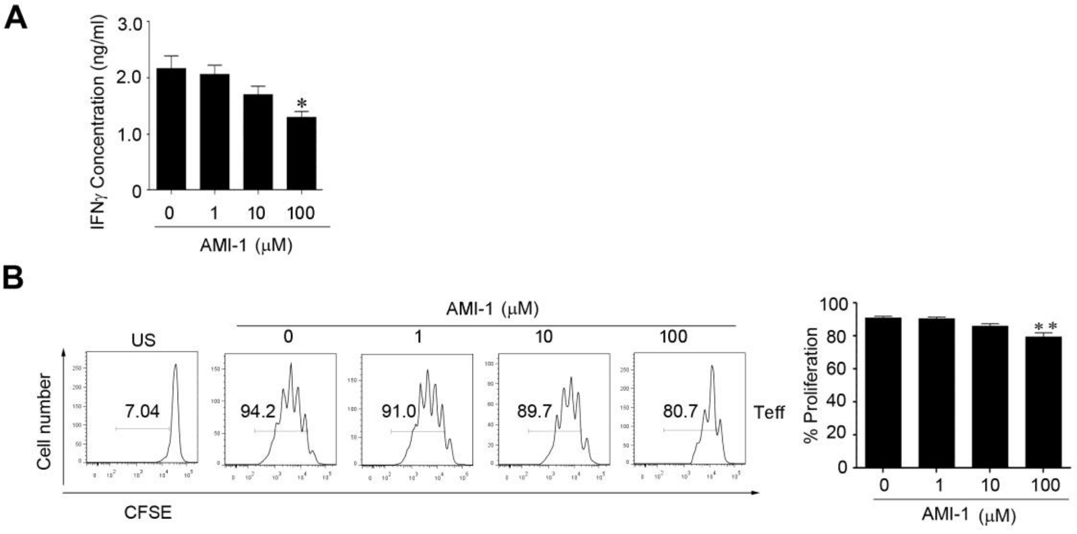

Supplementary Fig. 2: AMI-1 down-regulates IFN-γ secretion and cell proliferation in

$CD4^+$ T cells. $CD4^+$ T cells were treated with different AMI-1 concentrations and plated on anti-CD3 mAb-coated plates. After 48 h, the IFNγ levels in the supernatants were determined with an ELISA (A). Additionally, the cellular proliferation level was assayed with CFSE after 96 h (B). A summary of three independent experiments is presented. The data shown are means ± SEM in the right panel. The asterisks represent statistical significance between the groups, *P<0.05; **P<0.01.

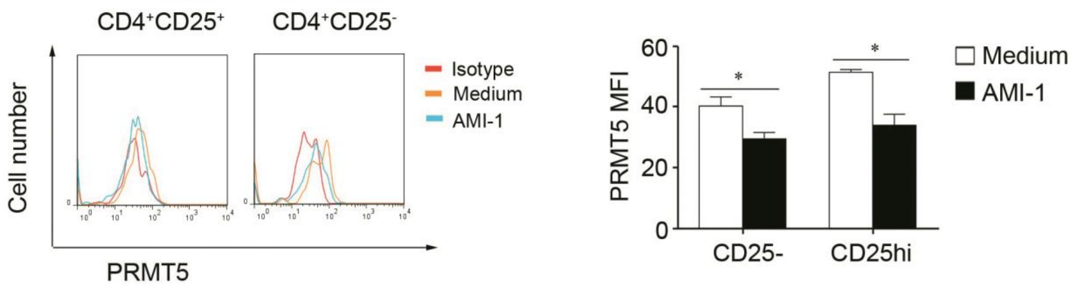

Supplementary Fig. 3: Treg and Teff cells were or were not treated with AMI-1 and analyzed with flow cytometry using antibodies specific for PRMT5 or an isotype control. The results are representative of three separate experiments. The *P* values represent statistical differences between the AMI-1-treated cells and the PBS-treated cells; *P<0.05.

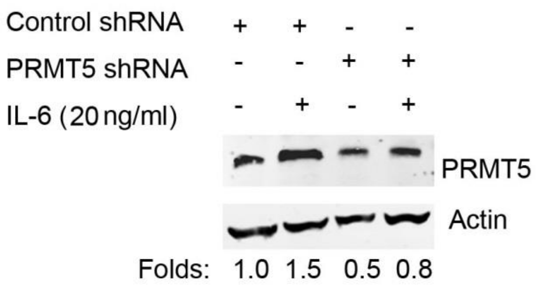

Supplementary Fig. 4: Tregs were sorted and electrophoretically transfected with mouse PRMT5 shRNA and treated with IL-6 20ng/ml or not. After 48 h, the PRMT5 expression level was measured using western blot analysis. Actin was utilized as the loading control.

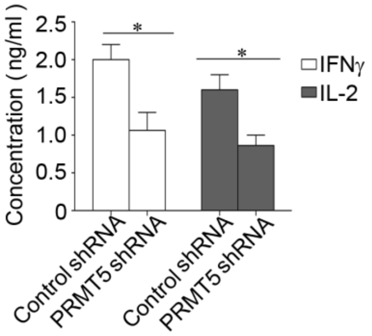

Supplementary Fig. 5: Reduction in PRMT5 protein levels by specific shRNA down-regulates IFNγ secretion in $CD4^+$ T cells. $CD4^+$ T cells were transfected with shRNA targeting PRMT5 or with control shRNA and plated onto anti-CD3 mAb-coated plates. After 48 h, the PRMT5 expression level was measured with western blot (A) analysis and the IFNγ and IL-2 levels in the supernatants were determined with ELISAs (B). A summary of three independent experiments is presented.

Supplementary Table 1. Mouse primers for q-PCR

Gene	Forward primer (5' to 3')	Reverse primer (5' to 3')
β-actin	TGTCCACCTTCCAGCAGATGT	AGCTCAGTAACAGTCCGCCTAGA
PRMT1	TACTACTTTGACTCCTATGCCCA	ATGCCGATTGTGAAACATGGA
PRMT2	AGCGCCGAGAAAGACTACC	GGCCTTGAAAAGAACTCCTTGA
PRMT3	CAGAGCACCAAAACACACTGG	TCAGGGTCACAATGAGGGAAC
PRMT4	ATCGCCCTCTACAGCCATGA	CTGTCTGCCCACACGACTG
PRMT5	CTGAATTGCGTCCCCGAAATA	AGGTTCCTGAATGAACTCCCT
PRMT6	GATGGGCTACGGACTTCTGC	GCATCTGGTCGCTAATCGGG-3
PRMT7	TTGCCAGGTCATCCTATGCC	GCCAATGTCAAGAACCAAGGC
PRMT8	ACGTGGTAGCAATCGAAGACA	GCTCCTTCATGGCAACATCC
PRMT10	CCTCTTCAGAATGGGCTTTCG	CCAGTTTGCTACGCGGTAAAA
Foxp3	GTGCCTGGTATATGCTCCCG	TAGGGTTGGGCATTGGGTTC
CTLA4	AACTGCAGCTGCCTTCTAGG	CTGCTAGCCAACACCACTGA
PD-1	AATGGCCTCTGTGGGTTCTG	CTCCCAAGGGTGGCTTTAGG
GITR	TGCCCAGCTATACCCTTGGT	CCGCTCTCATACACCCACTTC
